# CT-based subchondral bone and clinical predictors of long-term total ankle arthroplasty outcomes

**DOI:** 10.3389/fmed.2025.1713906

**Published:** 2026-01-12

**Authors:** Wei Ji, Deheng Liu

**Affiliations:** 1 Department of Joint Surgery, Qilu Hospital (Qingdao), Cheeloo College of Medicine, Shandong University, Qingdao, China; 2 Department of Hand and Foot Microsurgical Reconstruction Surgery, Qilu Hospital (Qingdao), Cheeloo College of Medicine, Shandong University, Qingdao, China

**Keywords:** gradient boosting model, random forest model, subchondral bone, support vector machine model, total ankle arthroplasty

## Abstract

**Objective:**

This study aimed to develop a machine learning-based predictive model for personalized long-term prognosis assessment in patients undergoing total ankle arthroplasty (TAA) by integrating preoperative computed tomography (CT)-derived subchondral bone structural parameters with clinical indicators.

**Methods:**

A retrospective cohort study involving 340 TAA patients was divided into training (*n* = 238, 70%) and validation (*n* = 102, 30%) sets through stratified random sampling, ensuring the outcome distribution was preserved. Radiographic features and clinical metrics were systematically collected. Univariate analysis was conducted to identify variables associated with poor prognosis in the training set, followed by feature reduction using the least absolute shrinkage and selection operator (LASSO) regression. To determine independent risk factors, multivariable COX proportional hazards regression (Cox regression) was used. Three machine learning models—Random Forest (RF), Support Vector Machine (SVM), and Gradient Boosting (GB)—were constructed using Python 3.8.5. Model performance was evaluated using receiver operating characteristic (ROC) curve analysis.

**Results:**

Baseline characteristics showed no statistically significant differences between training and validation sets (*p* > 0.05). Univariate analysis indicated that subchondral bone mineral density (BMD), trabecular separation (Tb. Sp), talar tilt angle, Charlson Comorbidity Index (CCI), and preoperative talar necrosis volume were significantly associated with the need for prosthesis revision surgery. In the multivariable COX regression, Tb. Sp, talar tilt angle, and preoperative talar necrosis volume emerged as independent risk factors for sustained clinical deterioration. Conversely, subchondral BMD and CCI were identified as protective factors. In the validation set, the area under the ROC (AUC) for the RF, SVM, and GB models was 0.897, 0.790, and 0.815, respectively. Pairwise comparisons using the DeLong test revealed a statistically significant difference in AUC between the RF and SVM models (ΔAUC = 0.107, *p* = 0.032) and between the RF and GB models (ΔAUC = 0.082, *p* = 0.041). In contrast, the difference between the SVM and GB models was not statistically significant (ΔAUC = 0.025, *p* = 0.597).

**Conclusion:**

The RF model that incorporates preoperative CT-quantified subchondral bone parameters and clinical indicators effectively predicts long-term adverse outcomes in TAA patients. The top three predictive features identified are subchondral BMD, Tb. Sp, and preoperative talar necrosis volume.

## Introduction

End-stage ankle osteoarthritis is a prevalent terminal ankle disorder in clinical practice, with its prevalence significantly increasing with age, affecting approximately 1–2% of the population over 60 years old (1, 2). This condition is characterized by persistent ankle pain and limited joint function, severely impairing patients’ quality of life. Many individuals struggle with walking and daily activities, such as climbing stairs or standing, and their symptoms often do not improve with conservative treatments (1). Radiographically, end-stage ankle osteoarthritis corresponds to Kellgren–Lawrence grade IV, which is also the most common indication for total ankle arthroplasty (TAA).

End-stage ankle osteoarthritis is defined by both radiographic and clinical criteria. The radiographic criteria include severe joint space narrowing (>50%), osteophyte formation, and subchondral sclerosis (2). The clinical criteria refer to persistent pain with a Visual Analogue Scale (VAS) score >50 mm for at least 6 months, which is unresponsive to conservative treatment (1). This condition aligns with the Kellgren–Lawrence grade IV classification. However, the long-term efficacy of TAA poses challenges, as complications such as prosthesis loosening, subsidence, and malalignment contribute to historically high failure rates, reaching 76% within 5 years (3).

Machine learning (ML) algorithms have demonstrated significant advantages in medical predictive modeling, particularly in handling high-dimensional, non-linear data. Random forest (RF) models have been successfully applied to predict geriatric syndromes and quality-of-life outcomes by capturing complex interactions among variables. In orthopedics, CT-based three-dimensional reconstruction and parametric measurements enable individualized assessments. In joint surgeries, ML models demonstrate superior performance compared to traditional methods, as they are capable of capturing non-linear variable interactions that traditional linear models fail to fully represent (4). In the context of TAA, previous studies relied solely on traditional linear models, identifying age and BMI as risk factors (5), without incorporating CT parameters. However, no study has systematically integrated CT-derived subchondral bone structural parameters with clinical indicators to develop a personalized predictive model for long-term TAA outcomes (4).

This study aimed to retrospectively analyze data from patients undergoing TAA by extracting subchondral bone parameters and clinical indicators. The goal was to construct a prognostic prediction model using machine learning (ML) algorithms. The findings are intended to enhance preoperative risk stratification, optimize surgical procedures, and support personalized interventions, ultimately improving long-term success rates for TAA.

## Materials and methods

### Study population

This retrospective study included 340 patients with end-stage ankle disorders who underwent TAA between June 2022 and June 2024. Sample size was determined through power analysis using G*Power 3.1 software. Based on Arshad et al.’s (5) retrospective study of TAA outcomes (*n* = 523), we set a significance level (*α*) of 0.05, a statistical power (1−*β*) of 0.8, and a medium effect size (*f*^2^ = 0.3), resulting in a minimum required total sample size of 280. Our study ultimately included 340 patients with end-stage ankle disorders, satisfying this sample size requirement. We used random sampling based on the primary outcome of favorable or poor status to divide the cohort into a training set (*n* = 238, 70%) and a validation set (*n* = 102, 30%). This stratification strategy ensured consistent outcome distributions across subsets, with 14.71% of the training set and 15.69% of the validation set presenting with poor outcomes. This approach prevents baseline imbalances and adheres to standard predictive modeling practices (4, 6). We compared the baseline characteristics of the two sets (Table 1) and found no significant differences (all *p* > 0.05), confirming valid randomization. The cohort of 340 patients, which includes 238 in the training set and 102 in the validation set, meets the required criteria. For the poor outcome group consisting of 51 patients, we implemented 10-fold cross-validation during model training to mitigate bias from small subgroup sizes, following the recommendations of Rennie et al. (4) for prognostic models with imbalanced outcomes.

**Table 1:** Comparison of baseline characteristics between training and validation sets.

Variables	Training set (*n* = 238)	Validation set (*n* = 102)	*t*/*χ*^2^	*P*
Age (years)	68.45 ± 9.23	67.89 ± 8.76	0.520	0.603
Gender (male/female)	131/107	59/43	0.227	0.634
BMI (kg/m^2^)	28.67 ± 4.12	29.01 ± 4.35	0.686	0.493
Smoking history (yes/no)	78/160	30/72	0.372	0.542
Diabetes (yes/no)	62/176	29/73	0.207	0.650
Rheumatoid arthritis (yes/no)	45/193	22/80	0.320	0.572
Subchondral bone density (HU)	412.35 ± 118.46	425.81 ± 121.03	0.954	0.341
Bone volume fraction (%)	18.23 ± 5.67	17.89 ± 5.41	0.514	0.608
Trabecular thickness (mm)	0.21 ± 0.07	0.22 ± 0.06	1.258	0.209
Trabecular separation (mm)	1.05 ± 0.31	1.08 ± 0.29	0.833	0.405
Subchondral cyst volume (mm^3^)	145.67 ± 85.34	152.93 ± 89.17	0.709	0.479
Bone defect area (mm^2^)	35.78 ± 15.69	37.45 ± 16.22	0.890	0.374
Sclerotic bone region ratio (%)	22.45 ± 8.91	23.11 ± 9.23	0.619	0.536
Talar tilt angle (°)	5.67 ± 2.34	5.89 ± 2.41	0.787	0.432
Hindfoot moment arm (mm)	12.34 ± 3.56	12.01 ± 3.78	0.769	0.443
Articular surface roughness (μm)	285.91 ± 105.67	275.43 ± 98.54	0.855	0.393
ASA classification (I/II/III/IV)	32/135 / 65/6	15/55 / 28/4	0.659	0.883
Charlson Comorbidity Index	3.56 ± 1.89	3.72 ± 1.95	0.709	0.479
Concomitant osteotomy (yes/no)	89/149	42/60	0.431	0.511
Soft tissue balancing procedure (yes/no)	121/117	48/54	0.408	0.523
Operative time (min)	142.56 ± 35.78	138.90 ± 33.45	0.881	0.379
Preoperative ankle range of motion (°)	28.56 ± 10.23	27.89 ± 9.87	0.559	0.576
Lower limb malalignment (yes/no)	101/137	47/55	0.385	0.535
Preoperative talar necrosis volume (mm^3^)	1250.45 ± 450.38	1315.67 ± 465.29	1.212	0.227
Preoperative PROMIS physical function score	35.67 ± 8.91	34.89 ± 9.23	0.732	0.465

Rheumatoid arthritis refers to a history of rheumatoid arthritis in remission (not active) at the time of enrollment; active rheumatoid arthritis was excluded per inclusion/exclusion criteria.

This retrospective study was approved by the Ethics Committee of Qilu Hospital of Shandong University in Qingdao, with Approval Number QL-2022-02-15 and Approval Date 15 February 2022. All patients provided written informed consent prior to surgery, permitting the retrospective use of their clinical and imaging data for research purposes and the publication of de-identified study results.

### Inclusion criteria

Diagnosis of end-stage ankle osteoarthritis (Kellgren–Lawrence grade IV) has been established due to unsuccessful conservative management. This is characterized by a persistent Visual Analogue Scale (VAS) pain score >50 mm and an AOFAS Ankle-Hindfoot Score <60. These conditions persist despite 12 weeks of physical therapy, 3 months of oral non-steroidal anti-inflammatory drugs (NSAIDs), and up to three intra-articular corticosteroid injections administered 4 weeks apart. This diagnosis is made in accordance with the 2021 Chinese Osteoarthritis Guidelines (2) and OARSI treatment response criteria (1). Patients underwent primary, unilateral TAA. Preoperative ankle CT scans and baseline clinical data are available, with ≥2 years of postoperative follow-up. Complete follow-up data, including radiographic and functional assessments. In this study, Kellgren–Lawrence grade IV, which indicates end-stage ankle osteoarthritis, is characterized by extensive joint space narrowing, clear osteophyte formation, subchondral sclerosis, and potential joint deformity (2).

### Exclusion criteria

Those with concurrent active rheumatoid arthritis, acute gouty arthritis, or other active inflammatory joint diseases. Severe cardiopulmonary, hepatic, or renal dysfunction, as well as systemic conditions that contraindicate surgery, are considered significant factors. Additionally, a history of prior ipsilateral ankle fusion, revision surgery, or joint infection poses further risks. Furthermore, patients who lost follow-up or lacked critical clinical/imaging data hinder the ability to assess outcomes accurately.

### Data collection

To ensure a comprehensive outcome assessment, data were collected at two distinct time points: preoperative baseline and postoperative follow-up. Preoperative baseline assessments were conducted 1 week before surgery, while postoperative follow-up evaluations occurred at 3, 6, 12, and 24 months after surgery, with the 24-month follow-up designated as the primary time point for outcome classification. During the preoperative baseline phase, the research team collected various categories of information, including demographic data such as age, gender, body mass index, and smoking history. They also documented comorbidities and surgical history, which included diabetes, rheumatoid arthritis, the Charlson Comorbidity Index (CCI), American Society of Anesthesiologists (ASA) classification, and prior ankle surgeries, as well as preoperative ankle CT scans and patient-reported outcome measurements (PROMs). Postoperative follow-up data consisted of postoperative PROMs, radiographic assessments, and prosthesis revision status.

PROMs addressed three key areas: ankle function, pain evaluation, and quality of life. All data were collected using standardized questionnaires at specified follow-up time points. Objective functional measures included preoperative ankle range of motion and the 6-min walking distance, assessed by trained medical staff using standard instruments.

Surgical parameters included operative time, concomitant osteotomy, and soft-tissue balancing procedures. All TAA procedures were performed by three senior orthopedic surgeons, each with over 10 years of clinical experience in ankle surgery. They adhered to a standardized surgical protocol developed in accordance with the 2021 Chinese Osteoarthritis Guidelines (2) and international TAA surgical consensus. This protocol ensured consistency in prosthetic selection criteria based on patient ankle anatomy and disease severity, used a transfibular surgical approach, and utilized standardized intraoperative soft-tissue balancing techniques, all aimed at maintaining uniformity across surgical operations.

CT-derived structural measurements were acquired using a Siemens Somatom Definition Flash scanner with the following parameters: slice thickness of 0.6 mm, voxel size of 0.3 × 0.3 × 0.6 mm^3^, acquisition protocol of 120 kV and 200 mAs, and an iterative reconstruction technique. These measurements fall into three core categories: subchondral bone quantitative parameters, morphological and alignment parameters, and a disease severity indicator. Subchondral bone parameters included bone density, represented by mean Hounsfield units, as well as bone volume fraction, trabecular thickness, trabecular separation, trabecular number, subchondral cyst volume, bone defect area, and the proportion of sclerotic regions. Morphological and alignment parameters encompassed the talar tilt angle, tibial plafond angle, and hindfoot alignment metrics, including the moment arm, tibiocalcaneal angle, and malalignment status. The disease severity indicator was defined as preoperative talar necrosis volume. All subchondral bone parameters were measured using Mimics 21.0 (Materialise, Belgium) and ImageJ 1.8.0 (NIH, USA) software, with detailed measurement procedures, software parameter settings, and representative CT images with segmentation annotations provided in Supplementary file 1 (Measurement Protocol for Subchondral Bone Parameters). Alignment parameters, such as the talar tilt angle and tibial plafond angle, were measured on preoperative CT images using Mimics 21.0 software. The talar tilt angle was defined as the angle between the horizontal line of the tibial plafond and the articular surface of the talus, while the tibial plafond angle was measured as the angle between the tibial shaft axis and the tibial plafond articular surface; comprehensive measurement protocols are provided in Supplementary material.

### Outcome definitions

Based on OARSI treatment response criteria, the 2021 Chinese Osteoarthritis Guidelines, and expert consensus on TAA, patients were stratified into two groups (2). The 24-month follow-up period was selected as the primary endpoint based on clinical guidelines and evidence indicating that most early-to-mid-term TAA complications, such as prosthesis loosening and subsidence, occur within this period (7, 8). This selection also facilitates comparisons with previous prognostic studies (9).

The classification of outcomes into favorable and poor groups is mutually exclusive and exhaustive, with no gray areas. A patient is classified as having a favorable outcome only if they meet all four criteria—clinical success, functional success, radiographic success, and freedom from revision—at the 24-month follow-up. Second, a patient is designated as having a poor outcome if any one of the following criteria is met at any point within the 24-month post-surgery: radiographic failure, revision surgery, or clinical failure. For example, a patient who achieves a visual analog scale improvement of at least 20 mm, thereby meeting the standard for clinical success, but also shows computed tomography-confirmed periprosthetic radiolucency greater than 2 mm, indicating radiographic failure, is classified as having a poor outcome. This example illustrates that there are no overlapping or ambiguous cases in the grouping system.

While historical 5-year failure rates provide insights into long-term outcomes, recent studies reveal that 60–70% of critical mid-term complications, including early loosening and subsidence, occur within 2 years postoperatively (7). This finding aligns with guidelines such as the 2021 Chinese Osteoarthritis Guidelines (2) and the Osteoarthritis Research Society International (OARSI) consensus (1), which recommend a 2-year follow-up as a core endpoint for mid-term TAA outcome assessment. This approach ensures the identification of modifiable early risks while maintaining comparability with contemporary studies (8, 9).

### Statistical analysis

Missing data were addressed using rigorous methodologies to minimize potential bias, with specific strategies implemented as follows. First, baseline data, including subchondral bone parameters and the Charlson Comorbidity Index—exhibiting an overall missing rate of 3.5%, corresponding to 12 cases out of 340 total participants—were imputed utilizing multiple imputation with five datasets generated through the mice package in R, a method that effectively accounts for inherent uncertainty in missing values (4). Second, for follow-up data, 12 patients, representing 3.3% of the cohort who were lost to follow-up, were censored at their last documented follow-up date, with the median time point being 9 months postoperatively. The Kaplan–Meier method was subsequently applied to incorporate these cases into survival analyses, a practice that aligns with the fundamental assumptions of Cox proportional hazards regression (5). Third, a sensitivity analysis was conducted to compare key baseline characteristics, including age, body mass index, and subchondral bone density, between the lost-to-follow-up and completed-follow-up patient groups. The results indicated no statistically significant differences across all measured metrics, with all *p*-values > 0.05, thereby confirming that bias resulting from loss to follow-up was minimal.

In the training set, a two-step variable selection approach was used to identify predictors. First, univariate screening was conducted with a threshold of *P* less than 0.05 to exclude variables that showed no significant association with the study outcomes. This was followed by the application of the least absolute shrinkage and selection operator (LASSO) regression for additional feature reduction.

This dual-step strategy is well-supported by prior research: Tibshirani et al., the developers of LASSO regression (6), have noted that it enhances analytical efficiency for high-dimensional data. Arshad et al. (5), as well as Zhang et al. (9), have validated its applicability in prognostic studies focused on thoracic aortic aneurysm; and Rennie et al. (4) have confirmed that it reduces analytical bias for datasets with small-to-medium sample sizes. After selecting the variables, multivariable Cox regression was used to identify independent predictors of the study’s outcomes.

Before fitting multivariate logistic regression and machine learning models, all continuous variables—such as subchondral bone density, trabecular separation, and talar tilt angle—were standardized using z-score normalization, resulting in a mean of 0 and a standard deviation of 1. This process eliminated the interference of different measurement units with the model parameters. The RF, Support Vector Machine (SVM), and Gradient Boosting (GB) models were trained using 10-fold cross-validation. To evaluate the performance and stability of the RF model, we conducted two key analyses and developed a nomogram for intuitive visualization of prognosis. First, out-of-bag (OOB) error rate analysis was performed: the OOB error rate (proportion of incorrect predictions for samples excluded from bootstrap tree training) was calculated (4), and the relationship between decision tree count (0–500) and OOB error rate was plotted to identify the optimal tree number (at error rate stabilization). Second, variable importance was ranked using two metrics: mean decrease in accuracy and mean decrease Gini (6). The RF-based nomogram was developed in three steps: first, core features were screened via LASSO regression; second, the RF model was trained on these selected features; and third, the predictions were converted into a visual nomogram using R’s rms package.

Model discrimination was assessed using receiver operating characteristic (ROC) curves, with area under the curve (AUC) serving as the primary metric. A statistical comparison of model performance was performed using the DeLong test (via the pROC package in R 4.2.3) (10), which evaluates pairwise AUC differences while accounting for model correlation (i.e., using the same validation set). GraphPad Prism 9.0 was used for ROC curve visualization.

## Results

### Comparison of baseline characteristics in the training and validation sets

The training set (*n* = 238) included 203 (85.29%) favorable and 35 (14.71%) poor outcomes; the validation set (*n* = 102) had 86 (84.31%) and 16 (15.69%), respectively. No significant differences in baseline characteristics were observed (*p* > 0.05) (Table 1).

### Univariate analysis of factors influencing prosthesis revision surgery

Univariate analysis showed statistically significant differences (*p* < 0.05) between the favorable and poor prognosis groups in the training cohort concerning subchondral bone density, trabecular separation, talar tilt angle, CCI, and preoperative talar necrosis volume (Table 2). In this analysis, the dependent variable was defined as the composite ‘poor outcome’, which encompasses radiographic failure, revision surgery, and clinical failure, as outlined in the Methods section. This definition aligns with the criteria used in the subsequent multivariate and machine learning analyses.

**Table 2:** Univariate analysis of factors influencing poor outcomes in total ankle replacement (TAR) patients.

Variables	Favorable prognosis group (*n* = 203)	Poor prognosis group (*n* = 35)	*t*/*χ*^2^	*P*
Age (years)	67.12 ± 8.45	69.01 ± 8.95	1.212	0.227
Gender (male/female)	105/98	18/17	0.001	0.975
BMI (kg/m^2^)	28.7 ± 3.8	29.1 ± 4.3	0.564	0.573
Smoking history (yes/no)	65/138	13/22	0.356	0.551
Diabetes (yes/no)	53/150	9/26	0.002	0.961
Rheumatoid arthritis (yes/no)	38/165	7/28	0.032	0.858
Subchondral bone density (HU)	435.21 ± 102.34	355.35 ± 115.46	4.182	0.001
Bone volume fraction (%)	18.23 ± 5.67	17.80 ± 5.91	0.412	0.681
Trabecular thickness (mm)	0.22 ± 0.06	0.21 ± 0.07	0.888	0.376
Trabecular separation (mm)	0.99 ± 0.26	1.28 ± 0.33	5.843	0.001
Subchondral cyst volume (mm^3^)	142.30 ± 80.15	158.90 ± 95.67	1.099	0.273
Bone defect area (mm^2^)	35.23 ± 14.87	38.14 ± 17.95	1.036	0.301
Sclerotic bone region ratio (%)	22.16 ± 8.52	24.31 ± 9.89	1.346	0.180
Talar tilt angle (°)	5.05 ± 1.77	7.23 ± 2.54	6.268	0.001
Hindfoot moment arm (mm)	12.42 ± 3.47	12.01 ± 3.98	0.631	0.528
Articular surface roughness (μm)	281.50 ± 100.25	275.80 ± 95.40	0.313	0.755
ASA classification (I/II/III/IV)	28/130 / 42/3	4/20 / 9/2	0.724	0.481
Charlson Comorbidity Index	3.03 ± 1.58	4.97 ± 1.16	6.943	0.001
Concomitant osteotomy (yes/no)	68/135	20/15	7.162	0.007
Soft tissue balancing procedure (yes/no)	103/100	18/17	0.006	0.940
Operative time (min)	141.54 ± 33.89	147.27 ± 38.53	0.905	0.366
Preoperative ankle range of motion (°)	28.91 ± 9.87	27.14 ± 10.56	0.970	0.333
Lower limb malalignment (yes/no)	78/125	21/14	5.721	0.017
Preoperative talar necrosis volume (mm^3^)	1150.21 ± 405.54	1725.87 ± 450.75	7.628	0.001
Preoperative PROMIS Physical Function score	36.14 ± 8.57	34.86 ± 9.23	0.807	0.421

multivariate and machine learning analyses.

### Multivariate cox regression analysis of factors influencing prosthesis revision surgery

Postoperative prognosis (dependent variable: favorable prognosis = 0, poor prognosis = 1) was analyzed using LASSO regression for variable selection (Supplementary Table 1). Variables with appropriate predictive power were included in the multivariate Cox regression analysis. The results indicated that trabecular separation, talar tilt angle, and preoperative talar necrosis volume were independent risk factors for a poor prognosis that necessitated prosthesis revision (*p* < 0.05). Additionally, subchondral bone density and the Charlson Comorbidity Index were identified as protective factors (*p* < 0.05) (Table 3).

**Table 3:** Multivariate logistic regression analysis of factors influencing prosthesis revision surgery.

Variables	β	SE	Wald	P	OR	95%CI
Subchondral bone density	−0.008	0.003	7.275	0.007	0.992	0.986 ~ 0.998
Trabecular separation	3.580	1.373	6.801	0.009	5.897	2.347 ~ 15.210
Talar tilt angle	1.409	0.339	17.275	0.001	4.093	2.106 ~ 7.954
Charlson Comorbidity Index	−1.265	0.364	12.059	0.001	0.282	0.138 ~ 0.576
Preoperative talar necrosis volume	0.003	0.001	13.927	0.001	1.003	1.001 ~ 1.004
Constant	−7.490	2.301	10.594	0.001	0.001	

### Predictive performance of machine learning models in training and validation sets

**Figure 1:**
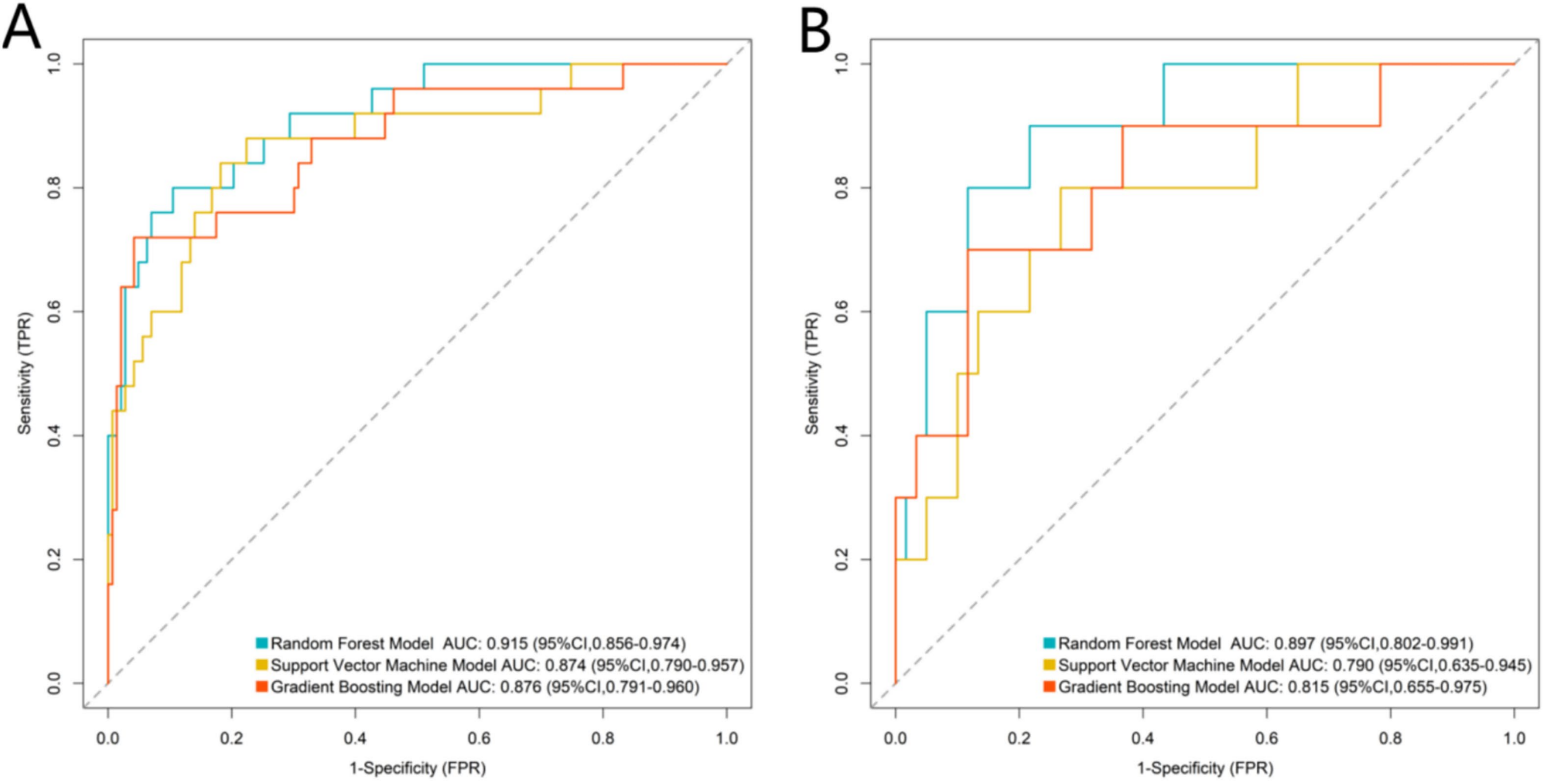
Receiver operating characteristic (ROC) curves of machine learning models (**A**: Training set and **B**: Validation set).

Based on the above five independent influencing factors, RF, SVM, and GB models were evaluated. The AUC values for the RF, SVM, and GB models in the validation set were 0.897 (95%CI: 0.802–0.991), 0.790 (95%CI: 0.635–0.945), and 0.815 (95%CI: 0.655–0.975), respectively. A DeLong test was conducted to compare the AUCs, revealing that the RF model’s AUC was significantly higher than that of the SVM (*p* = 0.032) and the GB model (*p* = 0.041). Consequently, the RF model was selected as the optimal model (Figure 1). To assess the complementary value of subchondral bone features, we developed a clinical-only RF model incorporating age, BMI, CCI, and other clinical indicators. This clinical-only model achieved an AUC of 0.723 (95%CI: 0.601–0.845) in the validation set, which was significantly lower than the combined model’s AUC of 0.897, as indicated by the DeLong test (*p* = 0.028). This finding confirms that subchondral bone features provide supplementary predictive information beyond clinical indicators.

### Development of a predictive model for prosthesis revision in TAR patients

**Figure 2:**
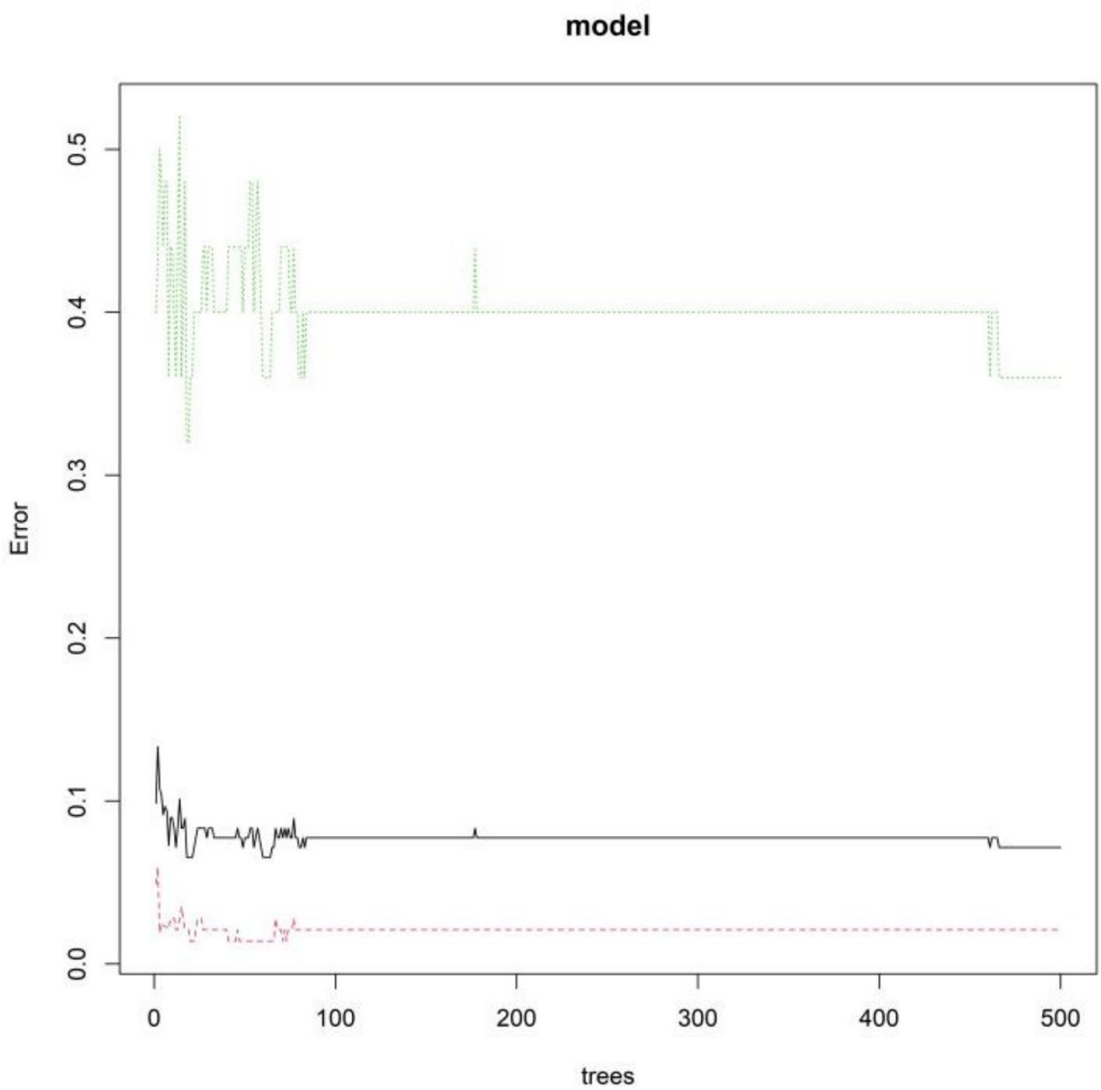
Out-of-bag (OOB) error rate and the number of decision trees in the random forest (RF) model. The *x*-axis represents the number of decision trees (ranging from 0 to 500), and the *y*-axis represents the OOB error rate (ranging from 0 to 0.5).

**Figure 3:**
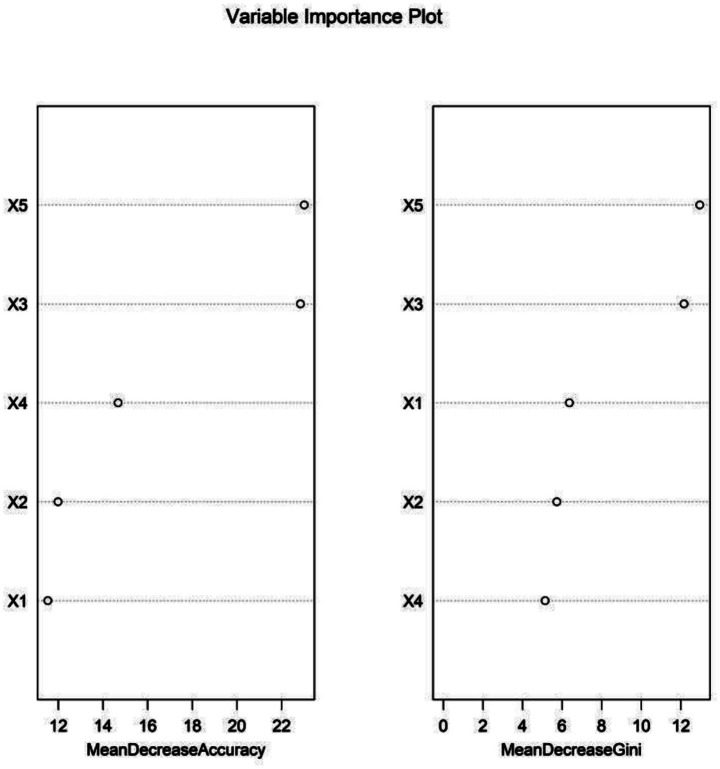
Variable importance ranking in the random forest (RF) model. X1 = subchondral bone density, X2 = trabecular separation, X3 = talar tilt angle, X4 = Charlson Comorbidity Index, and X5 = preoperative talar necrosis volume.

The RF model ranked the significance of the independent predictors as follows: preoperative talar necrosis volume, talar tilt angle, CCI, trabecular separation, and subchondral bone density (Figures 2, 3). OOB error rate stabilized at ~0.12 when the number of trees exceeded 200, which indicated that 200 is the optimal number of trees for the RF model (Figure 2). Figure 3 shows that preoperative talar necrosis volume, talar tilt angle, and CCI were the top three important features based on both mean decrease accuracy and mean decrease Gini metrics.

### Nomogram model based on five prognostic feature

**Figure 4:**
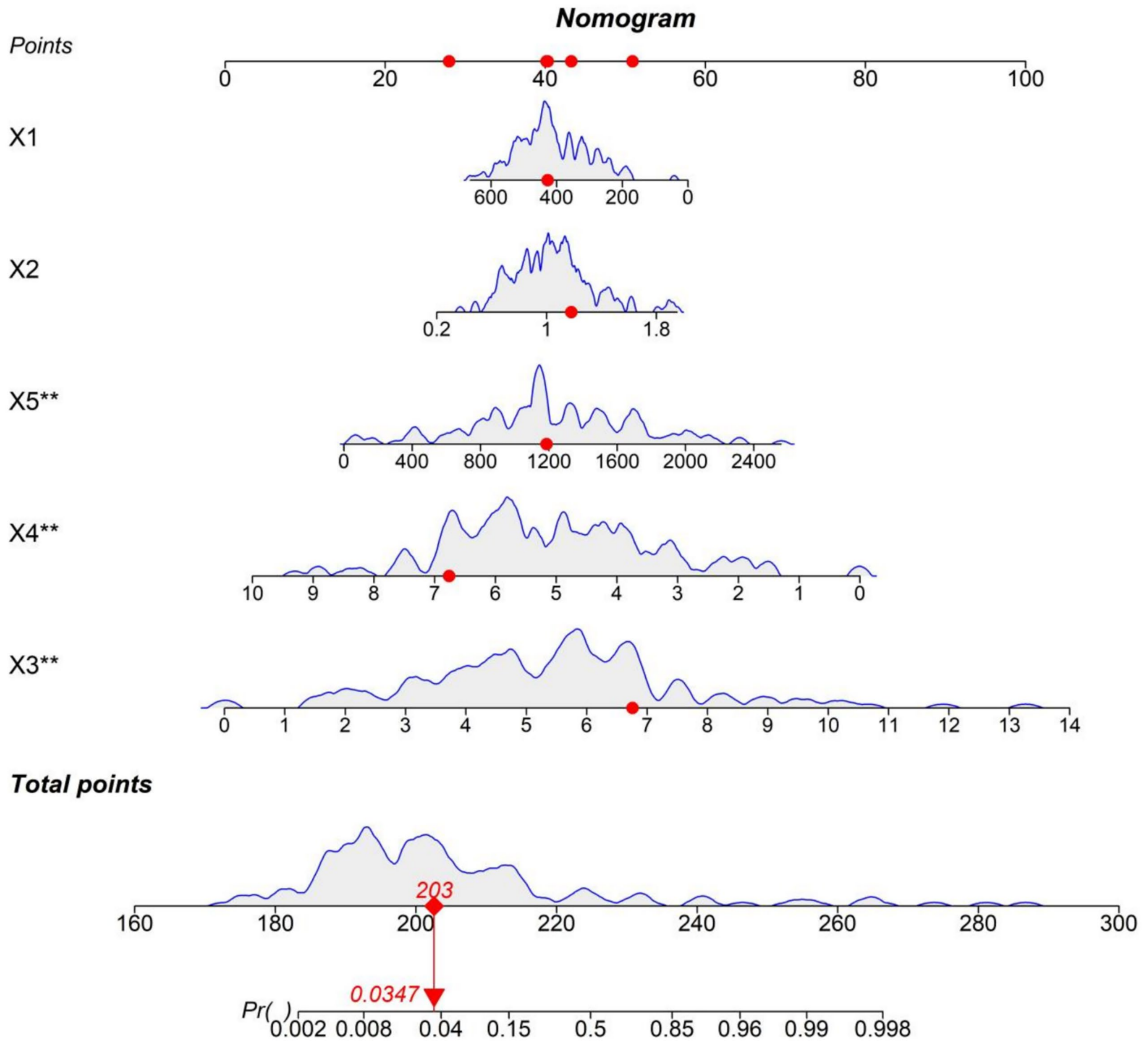
Nomogram based on the prognostic model. X1 = subchondral bone density, X2 = trabecular separation, X3 = talar tilt angle, X4 = Charlson Comorbidity Index, and X5 = preoperative talar necrosis volume. All other figures, tables, and their legends are correct. The image resolution meets publication standards.

A nomogram model was constructed to intuitively illustrate the impact of various features on prognosis. Trabecular separation, talar tilt angle, and preoperative talar necrosis volume are identified as risk factors, whereas subchondral bone mineral density and CCI serve as protective factors (Figure 4). Specifically, increased trabecular separation indicates a deterioration of bone microstructure, a larger talar tilt angle suggests joint instability, and an expanded preoperative talar necrosis volume compromises bone support capacity. In contrast, subchondral bone mineral density improves the stability of the prosthesis–bone interface, and effective management of CCI mitigates the influence of systemic factors on prognosis.

## Discussion

Recent advancements in prosthesis design and surgical techniques for TAA have made this procedure an effective treatment for end-stage ankle diseases, greatly enhancing patients’ quality of life. However, mid- to long-term complications such as prosthesis loosening, subsidence, and malalignment continue to be the primary causes of surgical failure and the need for revision, presenting significant challenges in clinical management (7, 8). To address these issues, we conducted a retrospective analysis of complete clinical data and high-resolution CT imaging from 340 TAA patients. Utilizing LASSO regression for variable selection and multivariate COX regression, we identified trabecular separation, talar tilt angle, preoperative talar necrosis volume, subchondral bone density, and the Charlson Comorbidity Index as independent predictors of mid- to long-term TAA outcomes.

Based on these key indicators, we developed three machine learning prediction models: RF, SVM, and GB. The results indicated that the RF model exhibited the best discriminative performance in both training and validation sets (AUC = 0.915), significantly outperforming the other two models. In the RF model, trabecular separation, subchondral bone density, and talar tilt angle were identified as the top three most important features. This suggests that abnormalities in subchondral bone microstructure, diminished bone quality, and ankle joint instability are critical factors influencing mid- to long-term TAA failure.

These findings provide valuable theoretical and data-driven support for preoperative risk assessment, individualized surgical planning, and postoperative management strategies (9). Our supplementary analysis further confirmed the importance of subchondral bone parameters. The clinical-only model exhibited a relatively low AUC of 0.723, whereas the combined model, which incorporated subchondral bone features, demonstrated significantly improved performance (*p* = 0.028). This indicates that subchondral bone parameters serve as valuable local structural biomarkers that complement clinical variables. They reflect the biomechanical integrity of the ankle joint, thereby enhancing the model’s predictive comprehensiveness.

Trabecular separation is a key microstructural parameter for evaluating bone quality. Increased trabecular separation indicates heightened bone resorption and diminished connectivity within the trabecular network, which directly contributes to a reduced mechanical strength of subchondral bone. Under normal conditions, trabeculae maintain a plate-like structure that forms a robust supporting network. In contrast, conditions such as osteoporosis or osteoarthritis cause this plate-like structure to gradually transition into a rod-like morphology, with some of these rod-like structures fracturing, leading to widened trabecular spacing (10). These microstructural changes have significant implications for TAA outcomes. First, increased trabecular separation compromises the initial stability of the prosthesis, raising the risk of micromotion and subsidence. Second, sparse trabecular structures hinder bone ingrowth, which diminishes the effectiveness of biologic fixation—porous tantalum prostheses, despite their excellent osseointegration potential, still rely on the quality of host bone. Finally, poor trabecular architecture accelerates stress shielding, resulting in progressive periprosthetic bone loss (11, 12).

Subchondral bone density emerged as a protective factor, ranking second in importance. Higher bone density is indicative of better mechanical properties of subchondral bone, which provides more stable support for the prosthesis (13). This finding corresponds with biomechanical studies demonstrating that reduced bone density significantly increases micromotion at the prosthesis–bone interface and exacerbates stress shielding, ultimately leading to loosening. Additionally, the talar tilt angle, a critical indicator of ankle joint stability and alignment, showed strong predictive value in this study (14). An increased talar tilt angle typically indicates joint instability or malalignment, which subjects the prosthesis to abnormal biomechanical loads, accelerating polyethylene liner wear and loosening.

The talar tilt angle is an essential parameter for assessing ankle stability and alignment and was confirmed in this study as an independent predictor of TAA outcomes. Under normal conditions, the talar tilt angle should not exceed 5°; an increased angle suggests joint instability or malalignment, resulting in abnormal biomechanical loading on the prosthesis (15). Biomechanically, an elevated talar tilt angle leads to uneven load distribution, which accelerates polyethylene wear. Computational simulations indicate that each 1° increase in the talar tilt angle raises pressure concentration at the medial edge of the tibial component by 18%, while reducing pressure at the lateral edge (16). This uneven load distribution not only results in asymmetric liner wear but also increases the risk of prosthesis loosening (17). Furthermore, an increased talar tilt angle is closely associated with ligamentous insufficiency, where medial ligament laxity or lateral ligament tightness can cause a talar valgus tilt, disrupting normal joint biomechanics (18).

The CCI was identified as a protective factor for TAA outcomes, consistent with findings from other joint arthroplasty studies. This underscores the importance of considering not only local factors but also patients’ systemic conditions when assessing TAA prognosis. The mechanisms by which comorbidities affect TAA outcomes can be summarized as follows: first, chronic conditions such as diabetes and cardiovascular diseases impair tissue healing and infection resistance, increasing the risk of postoperative complications. Studies show that diabetic patients experience 30–40% longer bone healing times and a 2.1-fold higher risk of infection compared to non-diabetic patients (19). Second, renal insufficiency and malnutrition disrupt bone metabolism, compromising osseointegration. Third, multiple comorbid conditions limit rehabilitation capacity, ultimately impairing functional recovery. Preoperative talar necrosis volume emerged as a strong predictor of TAA outcomes, reflecting disease severity and the mechanical integrity of the talus (20). Larger necrotic volumes typically reflect severe structural damage to subchondral bone, directly affecting initial prosthesis stability and long-term osseointegration. The mechanisms by which talar necrosis influences TAA outcomes primarily involve loss of mechanical support and reduced biological activity, as necrotic bone exhibits diminished mechanical strength and impaired osteogenic potential, which hinder bone ingrowth (21).

In this study, the RF model demonstrated superior predictive performance (AUC = 0.915), owing to its capacity to manage complex medical data effectively. Machine learning models, particularly RF, significantly excelled at capturing intricate non-linear relationships and interactions among CT-derived parameters and clinical variables. By utilizing bootstrap sampling and ensemble decision trees, the RF model reduced the influence of individual parameter fluctuations, thereby enhancing its robustness to data noise. Furthermore, the model’s feature importance ranking offered objectively quantified evidence for identifying key risk factors, transitioning from traditional single-parameter assessments to multidimensional predictive models. This shift ultimately improved prognostic accuracy and clinical applicability.

### Limitations

This study has four key limitations that need to be addressed. First, the retrospective single-center design may introduce selection bias. Among eligible cases, 89% of patients with complete clinical and imaging data demonstrated a higher follow-up compliance rate of 96%, compared to a compliance rate of 72% among patients with missing data. This discrepancy may lead to an overestimation of the model’s predictive accuracy. Additionally, information bias is present due to the lack of data on soft-tissue parameters, such as ligament laxity, which are known to influence TAA outcomes (18).

Second, the sample size for the poor outcome group is relatively small. While the total sample size (*n* = 340) meets the minimum requirement (*n* = 280) calculated by G*Power software, the poor outcome group includes only 35 patients in the training set and 16 patients in the validation set. Statistical simulations suggest that at least 100 poor outcomes are necessary to reliably detect rare events, such as periprosthetic fractures, which had an incidence of 1.2% in our cohort.

Third, CT imaging has inherent limitations, as it cannot assess cartilage quality—MRI T2 mapping is considered the gold standard for this purpose (21) or ligament integrity. Both cartilage quality and ligament integrity are crucial for implant stability but they were not evaluated in this study. Fourth, the follow-up period is relatively short. The 2-year endpoint captures only mid-term outcomes, leaving long-term risks, such as late prosthesis loosening after 5 years, unevaluated. Future studies should adopt a prospective multicenter design with a sample size exceeding 500 patients, integrate magnetic resonance imaging with computed tomography for a more comprehensive assessment, and extend the follow-up period to 5 years.

In conclusion, CT-based subchondral bone parameters such as trabecular separation and subchondral bone density, along with biomechanical indicators such as the talar tilt angle and preoperative talar necrosis volume, as well as clinical factors including the Charlson Comorbidity Index, are essential for predicting mid- to long-term TAA outcomes. Among the various machine learning models developed, the RF algorithm demonstrated outstanding predictive performance (AUC = 0.915). This study offers an effective tool for preoperative risk stratification, enhancing individualized outcome prediction by integrating CT imaging features with clinical parameters.

## Data Availability

The original contributions presented in the study are included in the article/Supplementary material, further inquiries can be directed to the corresponding author/s.
