# Supplementary material for: CT-based subchondral bone and clinical predictors of long-term total ankle arthroplasty outcomes
Source: Front Med (Lausanne). 2026 Jan 12;12:1713906. doi: 10.3389/fmed.2025.1713906 (PMC12832619; doi:10.3389/fmed.2025.1713906)
Supplement: Supplementary file 1 [file Data_Sheet_1.docx]

Supplementary File 1: Measurement Protocol for Subchondral Bone Parameters

1. Purpose

This file details the standardized protocol for measuring CT-derived subchondral bone parameters (e.g., bone mineral density, trabecular separation) to ensure reproducibility.

2. Equipment & Software

CT scanner: Siemens Somatom Definition Flash

Post-processing software: Mimics 21.0 (Materialise, Belgium), ImageJ 1.8.0 (NIH, USA) with BoneJ plugin

3. Step-by-Step Measurement Procedures

3.1 CT Image Import & Preprocessing

Import preoperative ankle CT images (DICOM format) into Mimics 21.0.

Adjust the image window (window width: 1500 HU, window level: 300 HU) to clearly visualize subchondral bone.

Crop the region of interest (ROI): Focus on the tibial plafond and talar dome subchondral bone (exclude soft tissues and non-target bone regions).

3.2 Subchondral Bone Segmentation

Use the "Thresholding" tool in Mimics to segment subchondral bone: Set HU range to 200–1500 (to exclude non-bone tissues [e.g., cartilage, cysts] and overly dense sclerotic bone).

Manually refine the segmentation mask: Erase non-target regions (e.g., subchondral cysts) and fill small gaps using the "Edit Mask" tool.

3.3 Parameter Calculation

Bone mineral density (BMD):

In Mimics, use the "Measure" tool to calculate the mean HU value within the segmented ROI—this value is used as BMD (unit: HU).

Trabecular microarchitecture parameters (trabecular separation [Tb.Sp], trabecular thickness [Tb.Th], bone volume fraction [BV/TV]):

Export the segmented ROI from Mimics as a TIFF file.

Import the TIFF file into ImageJ, then launch the BoneJ plugin.

Use "BoneJ Analyze" to calculate Tb.Sp (unit: mm), Tb.Th (unit: mm), and BV/TV (unit: %).

Preoperative talar necrosis volume:

In Mimics, segment the talar necrosis region (HU range: 50–200, distinct from normal bone).

Use the "Volume Calculation" tool to measure the volume (unit: mm³).

4. Quality Control

Inter-observer reliability: Two independent radiologists (with 5+ years of musculoskeletal imaging experience) measured 30 randomly selected cases—intraclass correlation coefficient (ICC) >0.90 for all parameters, confirming consistency.

Repeated measurement: The same observer re-measured 20 cases 1 week later—ICC >0.88, ensuring stability.

1. Representative Images


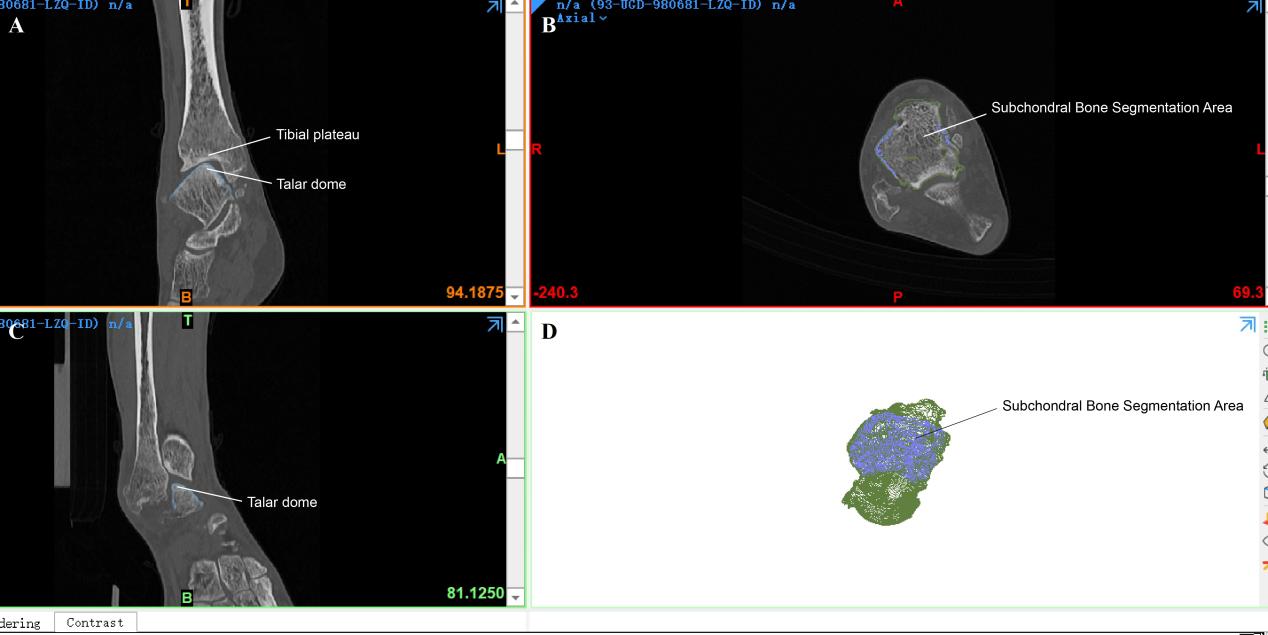


Figure S1-1: Original CT image of the ankle (tibial plafond and talar dome labeled) (A, B and C).

Figure S1-2: Segmented subchondral bone region in Mimics (D).

We have added the figure to give a better representation of the anatomy structures and the process of segmentation as required. In Figure S1-1 in particular, we have marked the Tibial plateau and Talar dome position in the original CT scans so that we can make sure they are anatomically correct. Moreover, in Figure S1-2, we marked the Subchondral bone segmentation area clearly to outline the specific area that was set in the process of segmentation.
