# Supplementary material for: CT-based subchondral bone and clinical predictors of long-term total ankle arthroplasty outcomes
Source: Front Med (Lausanne). 2026 Jan 12;12:1713906. doi: 10.3389/fmed.2025.1713906 (PMC12832619; doi:10.3389/fmed.2025.1713906)
Supplement: Supplementary file 2 [file Data_Sheet_2.docx]

Supplementary File 2: TRIPOD Checklist

| TRIPOD Item | Description | Corresponding Manuscript Section |
| --- | --- | --- |
| 1. Study type | Retrospective cohort study to develop a prognostic model | Study Population (Lines 88–89) |
| 2. Objectives | Develop a machine learning model to predict long-term TAA outcomes | Abstract (Objective), Introduction (Last Paragraph) |
| 3. Outcome | Composite "poor outcome" (radiographic failure, revision, clinical failure) | Outcome Definitions (Lines 183-207) |
| 4. Participants | 340 patients with end-stage ankle osteoarthritis who underwent TAA (June 2022–June 2024) | Study Population (Lines 88-111) |
| 5. Predictors | 5 key predictors: subchondral BMD, Tb.Sp, talar tilt angle, CCI, preoperative talar necrosis volume | Results (Univariate & Multivariate Analysis) |
| 6. Data sources | Clinical records, preoperative CT scans, postoperative follow-up data (X-ray/CT, PROMs) | Data Collection (Lines 132–149) |
| 7. Sample size | Calculated via G*Power 3.1 (minimum n=280); actual n=340 | Study Population (Lines 89-97) |
| 8. Missing data | Multiple imputation (baseline data) and Kaplan-Meier censoring (follow-up data) | Statistical Analysis (Lines 209-224) |
| 9. Variable selection | Univariate screening (P<0.05) + LASSO regression | Statistical Analysis (Lines 226-237) |
| 10.Model development | RF, SVM, GB models; 10-fold cross-validation | Statistical Analysis (Lines 238–254) |
| 11. Model validation | Internal validation (7:3 stratified random sampling) | Study Population (Lines 95-97) |
| 12.Model performance | AUC (RF: 0.897), DeLong test for model comparison | Results (Predictive Performance) |
| 13.Model calibration | Not applicable (binary outcome focus on discrimination) | Discussion (Limitations) |
| 14. Model updating | Not conducted (single-center data) | Discussion (Limitations) |
| 15.Model presentation | Python package + web calculator | Statistical Analysis (Last Paragraph) |
| 16. Clinical utility | Preoperative risk stratification for TAA patients | Discussion (4.4 Research and Clinical Implications) |
| 17. Ethics approval | Ethics Committee of Qilu Hospital (Qingdao) (No. QL-2022-02-15) | Study Population (Last Paragraph) |
| 18. Informed consent | Written informed consent from all patients | Study Population (Last Paragraph) |
| 19. Data availability | Available from corresponding author upon reasonable request | Availability of Data and Materials |
| 20.Conflicts of interest | None declared | Conflict of Interests |
| 21. Funding | Qingdao Key Health Discipline Development Fund (QDZDZK-2022095) | Funding Statement |
| 22.Protocol registration | Not applicable (retrospective study) | Clinical Trial Number |
